# The Relationship Between Health Parameters, Body Size, Elements of Lifestyle, and Hand Grip Strength in a Group of Patients with Type 2 Diabetes, Aged 40–98, from Ulaanbaatar, Mongolia

**DOI:** 10.3390/jcm14010102

**Published:** 2024-12-27

**Authors:** Myadagmaa Jaalkhorol, Agata Cieślik, Myagmartseren Dashtseren, Anya Khairat, Otgonbayar Damdinbazar, Gerelmaa Ochirdorj, Tsetsegsuren Khurelbaatar, Ganbayar Batmunkh, Ulemjjargal Ganzorig, Sławomir Kozieł

**Affiliations:** 1 Department of Health Research, Graduate School, Mongolian National University of Medical Sciences, Ulaanbaatar 14210, Mongolia; myadagmaa@mnums.edu.mn; 2 Mongolian Naran Society for Osteoarthritis and Musculoskeletal Health, Ulaanbaatar 14210, Mongolia; 3 Department of Anthropology, Ludwik Hirszfeld Institute of Immunology and Experimental Therapy, Polish Academy of Sciences, 53-114 Wrocław, Poland; slawomir.koziel@pwr.edu.pl; 4 Department of Family Medicine, School of Medicine, Mongolian National University of Medical Sciences, Ulaanbaatar 14210, Mongolia; myagmartseren@mnums.edu.mn (M.D.); amd19e018@gt.mnums.edu.mn (A.K.); 5 Department for Science and Technology, Mongolian National University of Medical Sciences, Ulaanbaatar 14210, Mongolia; otgonbayar@mnums.edu.mn; 6 Department of Epidemiology and Biostatistics, School of Public Health, Mongolian National University of Medical Sciences, Ulaanbaatar 14210, Mongolia; gerelmaa.o@mnums.edu.mn; 7 Department of Midwifery, School of Nursing, Mongolian National University of Medical Sciences, Ulaanbaatar 14210, Mongolia; tsetsegsuren@mnums.edu.mn; 8 Graduate School, Mongolian National University of Medical Sciences, Ulaanbaatar 14210, Mongolia; ganbayar.b@mnums.edu.mn (G.B.); pmm18d320@st.mnums.edu.mn (U.G.); 9 Department of Preclinical Sciences, Pharmacology and Medical Diagnostics, Faculty of Medicine, Wroclaw University of Sciences and Technology, 50-376 Wrocław, Poland

**Keywords:** hand grip strength, type 2 diabetes, blood pressure, cross-sectional study

## Abstract

**Background/Objectives:** Decreased muscle strength and lower hand grip strength (HGS) values are observed in patients with type 2 diabetes (T2D). This study aimed to present the values of hand grip strength as a valuable tool in T2D treatment monitoring in the context of body size and lifestyle elements in 347 patients with type 2 diabetes from Ulaanbaatar, Mongolia. **Methods:** A retrospective cross-sectional study was performed at hospitals in Ulaanbaatar, Mongolia. The maximum grip strengths of the right and left hands were measured three times, alternatively, using a digital hand dynamometer. The maximum grip strength of the dominant hand was used for the analysis. **Results:** The values of HGS in both hands dropped with increased age, systolic blood pressure (SBP), the duration of diabetes, and low glucose blood levels, whereas they increased with height. Patients who smoked had the lowest three values of HGS (the average value for each hand), whereas patients who quit smoking had the highest values. Second-order interactions between diastolic blood pressure (DBP) and sex showed a significant effect on the average HGS of both hands and for the left hand in particular (where HGS decreased only in females, whereas it increased in males). **Conclusions:** HGS is influenced by important socioeconomic and anthropometric factors in patients with type 2 diabetes, making it a valuable indicator of general health.

## 1. Introduction

Type 2 diabetes (T2D) is a metabolic disease of complex etiology characterized by chronic hyperglycemia with the abnormal metabolism of carbohydrates, fat, and protein due to defective insulin secretion and function within the human body. T2D (formerly known as non-insulin-dependent diabetes) results from an interaction between genetic, environmental, and behavioral risk factors [1]. The effects of diabetes include neuropathy, skin complications, eye complications, diabetic ketoacidosis, gastroparesis, and macrovascular diseases [2], and if left untreated, the disease carries a risk of increased mortality.

According to data published by the World Health Organization, about 422 million people worldwide have diabetes, with the majority living in low-and middle-income countries. In total, 1.5 million deaths are directly attributed to diabetes each year [3]. The progressively recorded increase in the incidence of T2D is mainly related to an unhealthy lifestyle leading to obesity, which is the main risk factor for type 2 diabetes. In recent years, this tendency has been particularly noticeable in developing countries, especially in Asia and Africa [1]. This is mainly due to the high price of healthy food, which increased significantly after the COVID-19 pandemic [4]. Secondly, poor dietary habits include primarily the consumption of ultra-processed foods (UPFs) [5], especially those rich in sugar and saturated fats [6]. Thirdly, a decreased level of physical activity also contributes. Since type 2 diabetes is not only a strictly medical problem but also constitutes an organizational and economic burden for the global healthcare system, extensive projects should be carried out to educate societies about a healthy lifestyle and the early detection of diabetes before serious health consequences occur.

Hand dysfunctions in T2D are relatively common and result from various mechanisms. One of the most common musculoskeletal complications in the hand is also known as diabetic cheiroarthropathy (DCA) or limited joint mobility (LJM). This diabetic stiff hand syndrome is seen in type 1 and 2 patients [7]. This pathological condition is caused by non-enzymatic collagen glycation, leading to microvascular damage and polyarticular stiffness [8]. Other recognized afflictions are Dupuytren’s disease (DD), trigger finger (TF), and carpal tunnel syndrome (CTS) [7]. It is worth noting that hand dysfunctions, especially LJM, are associated with nephropathy, retinopathy, and neuropathy. Thus, it can be an early warning signal of the possible presence of one or more microvascular complications [7], so their quick detection may be crucial in diagnosing diabetes.

Impaired hand joint functions negatively affect muscle strength; thus, measuring hand grip strength (HGS), which is an effective measure of muscle strength [9], may be helpful in diabetes screening. HGS is easy to measure and has been validated for lower leg muscle function [10] and performance testing [11]. The relationship between a decrease in hand grip strength and an increase in the risk of T2D results directly from the disease’s etiopathogenesis, which decreases muscle strength, power, mass, and quality, predisposing older patients to fall [12]. Type 2 diabetes is believed to have an immune and inflammatory background. Moreover, hyperglycemia directly impacts the intrinsic properties of the muscle to generate force [13]. McDonald and coauthors [14] indicated that current dietary and metabolic health shifts associated with increased hyperglycemia might impair muscular and organismal adaptations to exercise training.

Decreased muscle strength and lower HGS values are observed in T2D. Data obtained from NHANES, USA (2011–2014) indicates the association between HGS, insulin resistance, and glucose metabolism in 959 adolescents (general, mixed population) [15]. Muscle strength is also inversely associated with the development of insulin resistance [16]. Li et al. [17] found an independent, inverse association between grip strength and arm muscle quality with incident T2D in men. Therefore, HGS may also be an adequate measure of the epidemiological status of T2D patients.

HGS could also be considered a valuable marker of general health and is particularly relevant to the aging population [18]. Kim et al. (2022) [19] presented an association between HGS and physical activity (PA) as well as fitness. According to the authors, HGS can estimate muscular strength and endurance, aerobic fitness, flexibility, balance skills, coordination skills, and the overall physical fitness (PF) level in older adults. This could be used as a substitute test for the PF level in certain situations [19]. The multidirectional relationship between low HGS and various medical problems is also well-recognized. Low HGS may indicate a higher risk of mortality from chronic cardiovascular diseases [20]. Other diseases that have shown a correlation with low HGS are stroke, liver disease, some cancers, sarcopenia, and fragility fractures [18].

Mongolia is a low-income, northeastern Asian country with a population of 3.3 million people [21]. It is following the trend of other Asian countries in terms of an increase in the prevalence of T2D [22]. Dayan et al. (2023) [21] showed a threefold increase in diabetes prevalence over the last twenty years. Moreover, the authors identified obesity, a lack of physical exertion, and high blood pressure as modifiable risk factors for diabetes. Furthermore, the need for further research to analyze the prevalence of diabetes and risk factors in rural nomadic populations where traditional red meat dietary patterns are maintained was emphasized by the authors [21]. Implementing a simple method to facilitate the effective diagnosis of increased hyperglycemia could be a useful epidemiological tool to evaluate the dynamics of T2D prevalence in Mongolian populations. The current study aims to present the values of hand grip strength as a valuable tool in T2D treatment monitoring in the context of body size and lifestyle elements in 347 (139 males and 208 females) patients with type 2 diabetes from Ulaanbaatar, Mongolia.

## 2. Materials and Methods

### 2.1. Study Design, Setting, Participants, and Questionnaire

We conducted a retrospective cross-sectional study at health centers in Ulaanbaatar, Mongolia. A total of 347 patients with T2D (139 men and 208 women) supervised by endocrinologists from 6 secondary-level healthcare service hospitals (Khan-Uul, Songinohairkhan, Sukhbaatar, Bayangol, Bayanzurh, Chingeltei) in Ulaanbaatar city participated in the study. The secondary level hospitals of Ulaanbaatar city provide specialized care such as emergency care, children’s disease treatment, internal medicine, endocrinologist, and neurologist services to the population of Ulaanbaatar city through health insurance. The study’s main criterion for inclusion was patients’ age (over 40). Additionally, the duration of diabetes (at least two years) and regular care by an endocrinologist from a diabetes clinic were considered. Trained nurses conducted detailed interviews using a structured questionnaire. All the participants completed the 20–30 min questionnaire on their background (education, marital status, employment, accommodation, smoking status, alcohol consumption, religion, and ethnicity) as well as clinical data (disease onset and duration, family history of diabetes, diabetic treatment, comorbidity from December 2022 to March 2023). The patient’s latest minimum fasting glucose level was determined using the T2D control card based on the most recent test in the patient’s medical record, as advised by the WHO guidelines. All the participants provided written informed consent before participating in the study. The study protocol was approved by the Ethics Committee of the Mongolian National University of Medical Sciences (MNUMS, No.: 2022/0/12–2023/D-04).

### 2.2. Measurement of Handgrip Strength

Handgrip strength was measured when standing with the arms straight down to the sides. The maximum grip strength of the right and left hands was measured three times alternatively using a digital hand dynamometer (digital grip strength dynamometer, T.K.K 5401; Takei Scientific Instruments Co., Ltd., Tokyo, Japan). After the HGS of both hands was measured, a 60 s resting interval was allowed. The maximum grip strength of the dominant hand was used for the analysis [23].

### 2.3. Statistical Analyses

Student’s *t*-test for independent samples was used in order to assess sex differences in the analyzed variables. Following this, in order to assess the relationships between HGS and other parameters, the generalized linear model was used with the logit link function. The model included the direct effect of each parameter and second-order interactions between particular parameters and sex. The model was separately applied to HGS for each hand and the average HGS of both hands. The significant effect of the second-order interaction is presented in the graphs. All calculations were conducted using Statistica 13.0 [24].

## 3. Results

The descriptive statistics of the analyzed characteristics in males and females are presented in Table 1. Males were significantly younger but had a longer duration of T2D than females. Females were the dominant sex among patients and accounted for nearly 60% of the sample. Almost 27% of patients still regularly smoked tobacco (15.9%) (chi-square = 31.65; *p* < 0.001) (Table 2).

HGS has shown significant relationships with age, height, blood pressure, years of diabetes, low blood glucose levels, and current smoking status (Table 3).

The values of HGS in both hands dropped with increased age, systolic blood pressure (SBP), years of diabetes, and low glucose blood levels, whereas they increased with height. Patients who smoked had the lowest three values of HGS (average and for each hand), but patients who quit smoking had the highest values.

**Figure 1:**
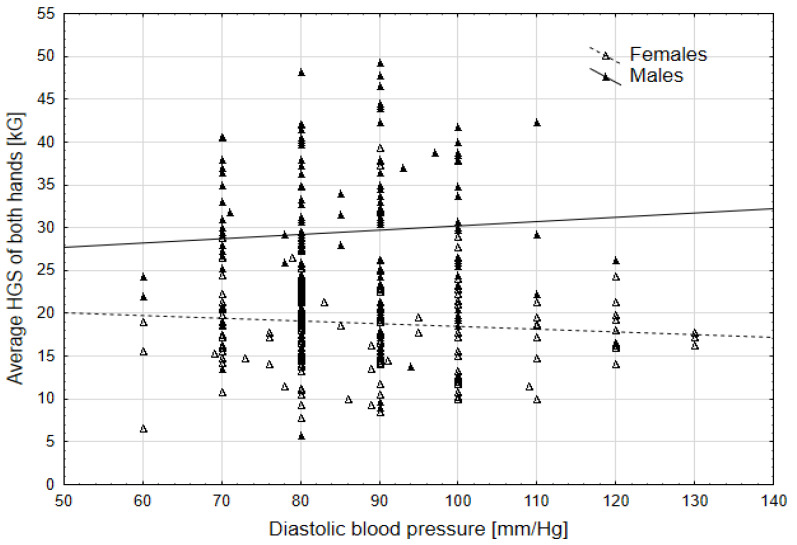
Relationship between the average HGS of both hands and DPB by sex.

**Figure 2:**
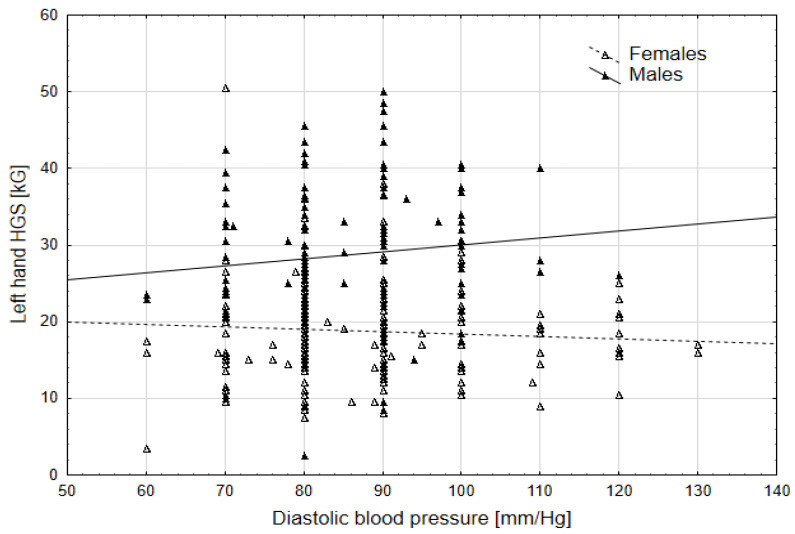
Relationship between the left HGS and DPB by sex.

The second-order interaction between diastolic blood pressure (DBP) and sex showed a significant effect on the average HGS of both hands and the left hand in particular. This means that with an increase in DBP, the values of HGS decreased, but only in females, whereas in males, there was an increase (Figure 1 and Figure 2). For all other parameters, the second-order interaction effects were not significant, indicating that significant parameters affected HGS values in the same manner in both sexes.

## 4. Discussion

The relationship between HGS, sociodemographic factors, and lifestyle has been extensively studied in different contexts and populations. In the present study, involving the Mongolian population from Ulaanbaatar, associations between the HGS of both hands and age, height, duration of diabetes, low glucose levels, DBP and SBP values, and current smoking habits were statistically significant. In contrast, a high level of glucose, BMI, and waist-to-hip ratio did not show any significant associations with HGS.

Among the sociodemographic factors, age was related to a decrease in hand grip strength, with grip strength decreased as age advanced [25]. Aging leads many elderly people to rely on visual feedback to compensate for poorer muscle strength in performing daily activities and preventing accidents [26]. Lin et al. (2014) [27] observed that aging reduces the maximum hand grip force output and the performance of bimanual coordination control of two hands, which may lead to difficulty with the execution of daily activities requiring both hands. The decline in neuromotor performance associated with the aging process of the nervous system can, therefore, be easily estimated using the HGS measurement. Moreover, it has been demonstrated that HGS may be considered a discriminating measure of neurological function and brain health [28].

Sex-related differences between males and females in HGS values are also reported in multiple studies, with men exhibiting a generally higher grip strength than women [25]. Bardo et al. (2021) [29] observed a significant effect of sex and hand dominance on grip strength but not on handedness, while hand shape and age had a more considerable influence on female grip strength. They also noted that females were significantly weaker with age, but grip strength was less affected in females with large hands than those with long hands. Females may also possibly experience a decline in HGS due to the menopausal transition [30].

HGS also shows an association with several anthropometric factors, with height exhibiting the strongest correlation [31]. Other anthropometric factors, such as body circumference measurements like arm circumference [31], hand length [32], palm width, and middle finger length [33], may affect hand grip strength. The association of HGS with an increase in body height, length, and arm circumference is probably related mainly to the greater muscle mass in taller and more athletic individuals. Research on athletes showed that HGS was significantly higher in male athletes, especially in their dominant hands, indicating a relationship between HGS and athletic performance [34].

Although absolute body weight was not assessed in our study, it is worth noting that this anthropometric variable may also show a close association with HGS. For instance, Rajesh et al. (2023) [35] noticed that in females, weight was the most strongly correlating factor with HGS, followed by BMI, while in males, hand span was the factor that most strongly correlated with HGS, followed by weight. Xu et al. (2023) [36], based on a cross-sectional study on 1511 healthy undergraduates, also indicated that weight has a significant positive correlation with hand grip strength and is more strongly correlated with hand grip strength than height. Additionally, several studies showed that the body mass index (BMI), which is closely related to weight, has been found to have a negative correlation with HGS, with a higher BMI associated with lower grip strength [37]. A weak association was observed between the dominant hand’s grip strength and BMI in Brazilian men [38]. In our study, based on the Mongolian population, there was no statistically significant association between BMI and HGS. However, the exact relationship between these variables remains a topic of ongoing research, especially in elderly age categories [39].

It is known that a high BMI is associated with obesity, which is considered an important predictor of T2D, resulting in decreased muscle strength and, therefore, reduced HGS values. As shown in several works of research, the duration of diabetes is negatively correlated with HGS. For instance, Ramhalo et al. (2023) [25] observed that a longer duration of T2D was linked to decreased hand grip strength, with a decrease of around 3 kg for every 10 years of the disease’s duration. Wu et al. (2022) [40] showed that a longer duration of T2D was associated with handgrip strength. Relative HGS was shown to be a better predictor of incident T2D. Hu et al. (2019) [41] observed that increased grip strength is independently associated with a lower prevalence of prediabetes in Chinese adults, suggesting that grip strength may be a useful marker for screening individuals at risk of prediabetes. The results of our study on a population in Mongolia are consistent with these observations, indicating that disease duration and chronic hyperglycemia lead to impaired muscle function and a decline in HGS.

Moreover, our study showed a statistically significant association between lower blood glucose levels and higher HGS, indicating that decreased glucose levels and controlled T2D positively influence the neuromuscular efficiency of the hand. Hyperglycaemia exerts detrimental effects on muscle physiology. Studies performed by Shannon et al. (2018) have shown that elevated glucose levels lead to insulin resistance, impairing glucose oxidation and altering metabolic glucose partitioning in skeletal muscle [42]. Hyperglycaemia also impairs myoblast proliferation, mitochondrial function, and muscle regeneration [43]; thus, uncontrolled diabetes may lead to lower HGS.

Another statistically significant association revealed in our study was between active smoking and hand grip strength. Overall, the link between smoking and decreased HGS is well known. Saito et al. (2012) [44] observed that cigarette smoking was associated with muscle strength in Japanese men; however, the authors could not identify the mechanism that links cigarette smoking and muscle strength [44]. Cigarette smoking, however, is usually associated with low levels of physical activity, a poor diet, and poorer general health. Smokers often have hormonal disorders, nutritional deficits, and lower levels of current and past leisure-time physical activity [45].

In our study, HGS was tested for both hands, with the left hand tested separately, and was also related to high values of both systolic and diastolic blood pressure (SBP, DBP). In the case of DBP, there was an interaction with sex, which means that as the value of systolic blood pressure increases, HGS decreases in females, while in males, it increases. The relationship between hand grip strength and DBP is complex and varies based on different factors. Chong et al. [46], in a Korean population, described that both relative and dominant hand grip strength showed a positive association with diastolic blood pressure among men aged 65–80 years, while in women aged 20–64, relative and dominant hand grip strength showed a positive relationship with diastolic blood pressure. Ji et al. (2018) [47] also observed an increased handgrip strength association with a higher DBP in men and women. The authors further concluded that in men, especially those who are overweight and obese, strong handgrip strength may be associated with a higher risk of hypertension [47]. A high DBP can influence decreased HGS in women, as indicated by various studies [48,49]. Lee et al. [49] highlighted that lower relative hand grip strength was linked to a higher risk of diabetes and impaired fasting glucose in women, emphasizing the impact of muscle strength on metabolic health. Furthermore, a study by Yang et al. (2021) [50] reported that women with decreased grip strength were more likely to experience nocturia, urgency, and incontinence, which are symptoms associated with high diastolic blood pressure. These findings underscore the intricate relationship between hand grip strength, blood pressure levels, and overall health outcomes in both males and females.

The relationship between grip strength and T2D is increasingly recognized, with studies indicating that grip strength may serve as a significant predictor of diabetes risk and related health outcomes. Grip strength is not only a measure of physical fitness but also correlates with metabolic health, particularly in individuals with T2D [51,52,53,54]. For this reason, HGS may be proposed as a cost-effective screening tool for diabetes, especially in resource-limited settings, underscoring its potential for more extensive use in diabetes management.

### Limitations

The present study has some limitations worth noting. Firstly, the participants were not selected randomly from different geographic areas in Mongolia; all the participants were Ulaanbaatar city citizens. Thus, this survey does not represent the general Mongolian population. Secondly, we did not obtain information regarding confounding factors, such as hyperglycemia and hypoglycemia, in this survey through blood tests but rather by interview. Additionally, the need for more detailed information on blood test measurements is a limitation that may have affected our ability to detect potential associations between these HGS and serum blood glucose levels. Third, this study is a cross-sectional study. Therefore, we did not measure the causal relationship between HGS- and T2D-related factors. However, it is worth mentioning that several cohort studies (e.g., [55,56]) have shown that HGS is associated with the course of diabetes. Fourth, we did not obtain more detailed information about clinical data, such as HbA1c in the blood. Therefore, future studies should consider collecting more comprehensive details through blood tests.

## 5. Conclusions

In this study, we have shown that in the Mongolian population from Ulaanbaatar, HGS is influenced by important socioeconomic and anthropometric factors in T2D patients of both sexes belonging to older age categories. Diabetes also shares risk factors with other global health threats, especially cardiovascular morbidity and mortality, hypertension, dyslipidemia, obesity, a lack of physical activity, and smoking, which are additional risk factors for CVD. T2D is also associated with chronic complications such as cardiovascular disease, cancer, infection, kidney disease, and diabetic coma, leading to increased morbidity and mortality. Our study, among others, showed a statistically significant association between lower blood glucose levels and higher HGS, indicating that decreased glucose levels and controlled T2D positively influence the neuromuscular efficiency of the hand. Using hand grip strength measurements as a supportive diagnostic tool can help monitor the effectiveness of diabetes treatment in T2D patients, which may be essential in middle- and low-income countries such as Mongolia. However, it should be underlined that other factors, such as age and the duration of diabetes, also play significant roles in muscle strength and overall health. Further research is needed to explore these relationships comprehensively.

## Figures and Tables

**Table 1:** Descriptive statistics of analyzed parameters by sex. Sex differences were assessed using Student’s *t*-test for independent samples.

	Males			Females			
Variables	N	Mean	SD	N	Mean	SD	t
Age [years]	139	58.55	7.96	208	60.75	9.45	2.26 *
High glucose [mmol/L]	139	18.14	8.07	208	18.32	6.61	ns
Low glucose [mmol/L]	139	7.46	3.72	208	7.98	3.17	ns
SBP [mm/Hg]	139	133.09	17.07	208	134.65	18.68	ns
DBP [mm/Hg]	139	85.15	10.71	208	87.35	13.73	ns
Years of diabetes	139	1.98	0.84	208	1.79	0.82	2.06 *
Height [cm]	139	168.96	6.36	208	160.65	5.92	12.43 **
BMI [kg/m^2^]	139	28.79	4.37	208	28.74	5.06	ns
Height-to-waist ratio	139	0.59	0.07	208	0.59	0.09	ns

* *p* < 0.05; ** *p* < 0.001.

**Table 2:** Distribution of sex and current smoking habits among patients.

Variables	N	%
Sex		
Male	139	40.06
Female	208	59.94
Current (every day) smoking		
Yes	93	26.80
No	254	73.20

**Table 3:** The results of the analysis of covariance were implemented using the generalized linear model with the binding logit function, where the HGS of both hands and average values were dependent variables, whilst age, sex, body size parameters, blood pressure, plasma blood glucose concentration, and the duration of T2D were independent variables. The model also included the effects of second-order interactions between sex and other health and lifestyle parameters.

	Average of Both Hands		Right Hand		Left Hand	
	Wald’s X^2^	*p*	Wald’s X^2^	*p*	Wald’s X^2^	*p*
Sex	0.08	ns	0.03	ns	0.15	ns
Current smoking	6.38	<0.05	5.24	<0.05	3.75	ns
Height	19.60	<0.001	12.19	<0.001	23.57	<0.001
BMI	0.50	ns	0.27	ns	0.66	ns
Waist-to-height ratio	2.69	ns	3.06	ns	1.89	ns
DBP	3.86	<0.05	1.65	ns	6.06	<0.05
SBP	8.11		5.26	<0.05	9.68	<0.01
High glucose	0.40	ns	0.19	ns	0.56	ns
Low glucose	10.11	<0.01	13.09	<0.001	5.58	<0.05
Years of diabetes	5.47	<0.05	5.30	<0.05	4.36	<0.05
Age	8.79	<0.05	6.61	<0.05	8.92	<0.01
	Interactions with sex					
DBP	4.96	<0.05	1.50	ns	9.15	<0.01
SBP	1.63	ns	0.30	ns	3.65	ns
High glucose	0.50	ns	1.14	ns	0.06	ns
Low glucose	0.06	ns	0.01	ns	0.30	ns
Years of diabetes	3.36	ns	2.42	ns	3.70	ns
Age	0.01	ns	0.00	ns	0.02	ns
Current smoking	0.01	ns	0.19	ns	0.39	ns

## Data Availability

The raw data supporting the conclusions of this article will be made available by the authors on request.

## References

[1] Wild S., Roglic G., Green A., Sicree R., King H. (2004). Global Prevalence of Diabetes: Estimates for the year 2000 and projections for 2030. Diabetes Care.

[2] Jacobson A.M., Braffett B.H., Cleary P.A., Gubitosi-Klug R.A., Larkin M.E., DCCT/EDIC Research Group (2013). The Long-Term Effects of Type 1 Diabetes Treatment and Complications on Health-Related Quality of Life: A 23-year follow-up of the Diabetes Control and Complications/Epidemiology of Diabetes Interventions and Complications cohort. Diabetes Care.

[3] World Health Organization Diabetes. https://www.who.int/health-topics/diabetes#tab=tab_1.

[4] Lewis M., Herron L.M., Chatfield M.D., Tan R.C., Dale A., Nash S., Lee A.J. (2023). Healthy Food Prices Increased More Than the Prices of Unhealthy Options during the COVID-19 Pandemic and Concurrent Challenges to the Food System. Int. J. Environ. Res. Public Health.

[5] Levy R.B., Rauber F., Chang K., da Costa Louzada M.L., Monteiro C.A., Millett C., Vamos E.P. (2021). Ultra-processed food consumption and type 2 diabetes incidence: A prospective cohort study. Clin. Nutr..

[6] Duan M.-J., Vinke P.C., Navis G., Corpeleijn E., Dekker L.H. (2022). Ultra-processed food and incident type 2 diabetes: Studying the underlying consumption patterns to unravel the health effects of this heterogeneous food category in the prospective Lifelines cohort. BMC Med..

[7] Pandey A., Usman K., Reddy H., Gutch M., Jain N., Qidwai S. (2013). Prevalence of hand disorders in type 2 diabetes mellitus and its correlation with microvascular complications. Ann. Med. Health Sci. Res..

[8] Persad-Paisley E.M., Lee C., Bhatt R.A. (2024). Understanding diabetic cheiroarthropathy: A focus on clinical presentation. J. Surg. Case Rep..

[9] Bohannon R.W., Magasi S.R., Bubela D.J., Wang Y.-C., Gershon R.C. (2012). Grip and knee extension muscle strength reflect a common construct among adults. Muscle Nerve.

[10] Martin H.J., Yule V., Syddall H.E., Dennison E.M., Cooper C., Aihie Sayer A. (2006). Is hand-held dynamometry useful for the measurement of quadriceps strength in older people? A comparison with the gold standard Bodex dynamometry. Gerontology.

[11] Visser M., Deeg D.J., Lips P., Harris T.B., Bouter L.M. (2000). Skeletal muscle mass and muscle strength in relation to lower-extremity performance in older men and women. J. Am. Geriatr. Soc..

[12] Maurer M.S., Burcham J., Cheng H. (2005). Diabetes mellitus is associated with an increased risk of falls in elderly residents of a long-term care facility. J. Gerontol. A Biol. Sci. Med. Sci..

[13] Orlando G., Balducci S., Bazzucchi I., Pugliese G., Sacchetti M. (2016). Neuromuscular dysfunction in type 2 diabetes: Underlying mechanisms and effect of resistance training. Diabetes Metab. Res. Rev..

[14] MacDonald T.L., Pattamaprapanont P., Pathak P., Fernandez N., Freitas E.C., Hafida S., Mitri J., Britton S.L., Koch L.G., Lessard S.J. (2020). Hyperglycaemia is associated with impaired muscle signalling and aerobic adaptation to exercise. Nat. Metab..

[15] Li S., Zhang R., Pan G., Zheng L., Li C. (2018). Handgrip strength is associated with insulin resistance and glucose metabolism in adolescents: Evidence from National Health and Nutrition Examination Survey 2011 to 2014. Pediatr. Diabetes.

[16] Grøntved A., Ried-Larsen M., Ekelund U., Froberg K., Brage S., Andersen L.B. (2013). Independent and combined association of muscle strength and cardiorespiratory fitness in youth with insulin resistance and β-cell function in young adulthood: The European Youth Heart Study. Diabetes Care.

[17] Li J.J., Wittert G.A., Vincent A., Atlantis E., Shi Z., Appleton S.L., Hill C.L., Jenkins A.J., Januszewski A.S., Adams R.J. (2016). Muscle grip strength predicts incident type 2 diabetes: Population-based cohort study. Metabolism.

[18] Vaishya R., Misra A., Vaish A., Ursino N., D’Ambrosi R. (2024). Hand grip strength as a proposed new vital sign of health: A narrative review of evidences. J. Health Popul. Nutr..

[19] Kim S.H., Kim T., Park J.-C., Kim Y.H. (2022). Usefulness of hand grip strength to estimate other physical fitness parameters in older adults. Sci. Rep..

[20] Shim J., Yoo H.J. (2020). Effects of Handgrip Strength on 10-Year Cardiovascular Risk among the Korean Middle-Aged Population: The Korea National Health and Nutrition Examination Survey 2014. Healthcare.

[21] Dayan A., Erkhembayar R., Luvsandavaajav O., Mukhtar Y., Enkhtuvshin B., Tumenbayar B. (2023). Prevalence of Type 2 Diabetes in Mongolia: Results from Population-Based Survey Compared with 1999 Study. Diabetes Metab. Syndr. Obes..

[22] Nanditha A., Ma R.C., Ramachandran A., Snehalatha C., Chan J.C., Chia K.S., Shaw J.E., Zimmet P.Z. (2016). Diabetes in Asia and the Pacific: Implications for the Global Epidemic. Diabetes Care.

[23] Chen L.-K., Woo J., Assantachai P., Auyeung T.-W., Chou M.-Y., Iijima K., Jang H.C., Kang L., Kim M., Kim S. (2020). Asian Working Group for Sarcopenia: 2019 Consensus Update on Sarcopenia Diagnosis and Treatment. J. Am. Med. Dir. Assoc..

[24] (2016). Dell Statistica (Data Analysis Software System).

[25] Ramalho D., Silva L., Almeida C., Rocha L., Rocha G., Veríssimo R. (2023). Hand grip strength: A reliable assessment tool of frailty status on the person with type 2 diabetes mellitus | La force de prehension: Un outil d’évaluation fiable de la fragilité chez la personne atteinte de diabète de type 2. Nutr. Clin. Metab..

[26] Lin C.-H., Sung W.-H., Chiang S.-L., Lee S.-C., Wang P.-C., Wang X.-M. (2019). Influence of aging and visual feedback on the stability of hand grip control in elderly adults. Exp. Gerontol..

[27] Lin C.-H., Chou L.-W., Wei S.-H., Lieu F.-K., Chiang S.-L., Sung W.-H. (2014). Influence of aging on bimanual coordination control. Exp. Gerontol..

[28] Carson R.G. (2018). Get a grip: Individual variations in grip strength are a marker of brain health. Neurobiol. Aging.

[29] Bardo A., Kivell T.L., Town K., Donati G., Ballieux H., Stamate C., Edginton T., Forrester G.S. (2021). Get a grip: Variation in human hand grip strength and implications for human evolution. Symmetry.

[30] Huebner M., Lawrence F., Lusa L. (2022). Sex Differences in Age-Associated Rate of Decline in Grip Strength When Engaging in Vigorous Physical Activity. Int. J. Environ. Res. Public Health.

[31] Byambaa A., Altankhuyag I., Damdinbazar O., Jadamba T., Byambasukh O. (2023). Anthropometric and Body Circumference Determinants for Hand Grip Strength: A Population-Based Mon-Timeline Study. J. Aging Res..

[32] Khader A., Almashaqbeh S. (2023). Handgrip Strength and its Association with Anthropometric Measurements at Different Anatomical Positions of Arm among Young Individuals. J. Biomim. Biomater. Biomed. Eng..

[33] Wey A.K.X., Caszo B.A., Ikram M.A., Gnanou J.V. (2023). Effect of hand anthropometry indices on the measurement of hand grip strength using a handheld dynamometer in young asian males. Eur. J. Phys. Educ. Sport Sci..

[34] Nivethapriya P., Neelambikai N., Shanmugavadivu R. (2018). Hand grip strength in athletes of different sports. Biomedicine.

[35] Rajesh M., Adithi H., Prathik P., Panth S.J., Deepak B.B.V.L., Bahubalendruni M.V.A.R., Parhi D.R.K., Biswal B.B. (2023). Statistical Study of the Influence of Anthropometric Parameters on the Hand Grip Strength of an Individual. Intelligent Manufacturing Systems in Industry 4.0. IPDIMS 2022.

[36] Xu T., Li X., Wang D., Zhang Y., Zhang Q., Yan J., Jiang J., Liu W., Chen J. (2023). Hand grip strength should be normalized by weight not height for eliminating the influence of individual differences: Findings from a cross-sectional study of 1511 healthy undergraduates. Front. Nutr..

[37] Salim S., Rose Davy C. (2023). Correlation of body mass index with handgrip strength and blood pressure indices among young adults. Indian J. Physiol. Pharmacol..

[38] De Andrade Fernandes A., Natali A.J., Vieira B.C., Do Valle M.A.A.N., Moreira D.G., Massy-Westropp N., Marins J.C.B.M. (2014). The relationship between hand grip strength and anthropometric parameters in men. Arch. Med. Del Deport..

[39] Soraya N., Parwanto E. (2023). The Controversial Relationship between Body Mass Index and Handgrip Strength in the Elderly: An Overview. Malays. J. Med. Sci..

[40] Wu H., Gu Y., Wang X., Meng G., Rayamajhi S., Thapa A., Zhang Q., Liu L., Zhang S., Zhang T. (2023). Association Between Handgrip Strength and Type 2 Diabetes: A Prospective Cohort Study and Systematic Review with Meta-analysis. J. Gerontol. A Biol. Sci. Med. Sci..

[41] Hu S., Gu Y., Lu Z., Zhang Q., Liu L., Meng G., Yao Z., Wu H., Bao X., Chi V.T.Q. (2019). Relationship Between Grip Strength and Prediabetes in a Large-Scale Adult Population. Am. J. Prev. Med..

[42] Shannon C., Merovci A., Tripathy D., Abdul-Ghani M., Norton L., Defronzo R.A. (2018). Effects of Hyperglycemia on Skeletal Muscle Glucose Metabolism in Healthy Subjects. Diabetes.

[43] Badu-Mensah A., Valinski P., Parsaud H., Hickman J.J., Guo X. (2022). Hyperglycemia Negatively Affects IPSC-Derived Myoblast Proliferation and Skeletal Muscle Regeneration and Function. Cells.

[44] Saito T., Miyatake N., Sakano N., Oda K., Katayama A., Nishii K., Numata T. (2012). Relationship between cigarette smoking and muscle strength in Japanese men. J. Prev. Med. Public Health.

[45] Szulc P., Duboeuf F., Marchand F., Delmas P.D. (2004). Hormonal and lifestyle determinants of appendicular skeletal muscle mass in men: The MINOS study. Am. J. Clin. Nutr..

[46] Chong H., Choi Y.E., Kong J.Y., Park J.H., Yoo H.J., Byeon J.H., Lee H.J., Lee S.H. (2020). Association of hand grip strength and cardiometabolic markers in Korean adult population: The Korea national health and nutrition examination survey 2015–2016. Korean J. Fam. Med..

[47] Ji C., Zheng L., Zhang R., Wu Q., Zhao Y. (2018). Handgrip strength is positively related to blood pressure and hypertension risk: Results from the National Health and nutrition examination survey. Lipids Health Dis..

[48] Anjakumar J.M., Shashikala L. (2023). Association between blood pressure and hand grip strength among adult population visiting a tertiary care centre—a cross-sectional study. Indian J. Appl. Res..

[49] Lee M.J., Khang A.R., Yi D., Kang Y.H. (2022). Low relative hand grip strength is associated with a higher risk for diabetes and impaired fasting glucose among the Korean population. PLoS ONE.

[50] Yang S.J., Park J.H., Oh Y., Kim H., Kong M., Moon J. (2021). Association of decreased grip strength with lower urinary tract symptoms in women: A cross-sectional study from Korea. BMC Womens Health.

[51] Park D.Y., Rho J.W., Kim E., Kim Y.S. (2024). Comparison of Absolute and Relative Grip Strength to Predict Incidence of Diabetes Mellitus in Korea: A Prospective Cohort Study. Metab. Syndr. Relat. Disord..

[52] Lu J., Wang Y., Shen Y., Mo Y., Ma X., Hu G., Zhou J. (2024). Potential modifying effect of grip strength on the association between glycated hemoglobin (HbA1c) and all-cause mortality in older adults with type 2 diabetes: Evidence from UK Biobank. Chin. Med. J..

[53] Wei L., Zeng J., Fan M., Chen B., Li X., Li Y., Xu S. (2024). Associations between handgrip strength and skeletal muscle mass with all-cause mortality and cardiovascular mortality in people with type 2 diabetes: A prospective cohort study of the UK Biobank. J. Diabetes.

[54] Nakanishi S., Shimoda M., Kimura T., Katakura Y., Sanada J., Fushimi Y., Iwamoto Y., Iwamoto H., Mune T., Kaku K. (2024). The impact of grip strength, waist circumference, and body mass index on Hemoglobin A1c value: Cross-sectional study using outpatient clinical data in Japanese elderly patients with type 2 diabetes mellitus. Geriatr. Gerontol. Int..

[55] Boonpor J., Parra-Soto S., Petermann-Rocha F., Ferrari G., Welsh P., Pell J.P., Sattar N., Gill J.M., Ho F.K., Gray S.R. (2021). Associations between grip strength and incident type 2 diabetes: Findings from the UK Biobank prospective cohort study. BMJ Open Diabetes Res. Care.

[56] van der Kooi A.L., Snijder M.B., Peters R.J., Van Valkengoed I.G. (2015). The association of handgrip strength and type 2 diabetes mellitus in six ethnic groups: An analysis of the HELIUS study. PLoS ONE.

